# Surface‐bound matrix metalloproteinase‐8 on macrophages: Contributions to macrophage pericellular proteolysis and migration through tissue barriers

**DOI:** 10.14814/phy2.14778

**Published:** 2021-03-03

**Authors:** Xiaoyun Wang, Duo Zhang, Quynh‐Anh Fucci, Clare M. Dollery, Caroline A. Owen

**Affiliations:** ^1^ Division of Pulmonary and Critical Care Medicine Brigham and Women's Hospital and Harvard Medical School Boston MA USA; ^2^ Center for Vaccines and Immunology University of Georgia Athens GA USA; ^3^ Clinical and Experimental Therapeutics College of Pharmacy University of Georgia and Charlie Norwood VA Medical Center Augusta GA USA; ^4^ Whittington Hospital Wittington Health NHS Trust London UK

**Keywords:** acute lung injury, chronic obstructive pulmonary disease, fibrosis, interstitial collagen, proteolysis

## Abstract

**Objective:**

MMP‐8 binds to surface‐bound tissue inhibitor of metalloproteinase‐1 (TIMP‐1) on PMNs to promote pericellular proteolysis during the development of inflammatory diseases associated with tissue destruction. Little is known about the biology of MMP‐8 in macrophages. We tested the hypotheses that: (1) MMP‐8 and TIMP‐1 are also expressed on the surface of activated macrophages, (2) surface‐bound MMP‐8 on macrophages promotes TIMP‐resistant pericellular proteolysis and macrophage migration through tissue barriers, and (3) MMP‐8 binds to surface‐bound TIMP‐1 on macrophages.

**Methods:**

Surface MMP‐8 and TIMP‐1 levels were measured on human monocyte‐derived macrophages (MDM) and/or murine macrophages using immunostaining, biotin‐labeling, and substrate cleavage methods. The susceptibility of membrane‐bound Mmp‐8 on activated macrophages from wild‐type (WT) mice to TIMPs was measured. Migration of WT and *Mmp*‐*8* ^−/−^ macrophages through models of tissue barriers in vitro and the accumulation of peritoneal macrophages in WT versus *Mmp*‐*8* ^−/−^ mice with sterile peritonitis was compared. Surface levels of Mmp‐8 were compared on activated macrophages from WT and *Timp*‐*1* ^−/−^ mice.

**Results:**

Lipopolysaccharides and a cluster of differentiation 40 ligand increased surface MMP‐8 and/or TIMP‐1 staining and surface type I collagenase activity on MDM and/or murine macrophages. Activated *Mmp*‐*8* ^−/−^ macrophages degraded less type I collagen than activated WT macrophages. The surface type‐I collagenase activity on WT macrophages was resistant to inhibition by Timp‐1. Peritoneal macrophage accumulation was similar in WT and *Mmp*‐*8* ^−/−^ mice with sterile acute peritonitis. However, *Mmp*‐*8* ^−/−^ macrophages migrated less efficiently through models of tissue barriers (especially those containing type I collagen) than WT cells. Activated WT and *Timp*‐*1* ^−/−^ macrophages had similar surface‐bound Mmp‐8 levels.

**Conclusions:**

MMP‐8 and TIMP‐1 are expressed on the surface of activated human MDM and murine macrophages, but Mmp‐8 is unlikely to bind to surface‐bound Timp‐1 on these cells. Surface‐bound MMP‐8 contributes to TIMP‐resistant monocyte/macrophage pericellular proteolysis and macrophage migration through collagen‐containing tissue barriers.

## Graphical abstract

Pro‐inflammatory mediators induce the expressed of matrix metalloproteinase‐8 (MMP‐8) on the the surface of monocytes and macrophages. Surface‐bound MMP‐8 on monocytes and macrophages is catalytically active but resistant to inhibition by tissue inhibitor of metalloproteinases‐1. Surface‐bound MMP‐8 on monocytes and macrophages contributes to the migration of these cells through tissue barriers containing type‐I collagen.

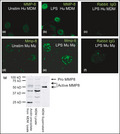

## 1 INTRODUCTION

Interstitial collagens are generally resistant proteolysis but can undergo initial cleavage by matrix metalloproteinase‐1 (MMP‐1), MMP‐8, MMP‐13, or membrane type‐1 MMP (MT1‐MMP), producing ¾ and ¼ fragments (Gross & Nagai, 1965) that are susceptible to further degradation by gelatinases (e.g., MMP‐2 and MMP‐9) (Owen & Campbell, 1999). Type I collagen is the most abundant collagen in skin, bone, and tendons but is also deposited in tissues during fibrotic reactions to injury (Craig et al., 2015). Although the substrate specificities of interstitial collagenases overlap, MMP‐8 degrades type I collagen three times more potently than MMP‐1 or MMP‐13 in vitro (Horwitz et al. 1977). The main activity for MMP‐8 (collagenase‐2 or neutrophil collagenase) was initially thought to be degradation of interstitial collagens. However, more recent studies have identified other activities for MMP‐8 including the processing of cytokines and chemokines to regulate inflammation and fibrosis (Balbin et al., 2003; Craig et al., 2013; Garcia‐Prieto et al., 2010; Quintero et al., 2010).

MMP‐8 is most highly expressed by PMNs (Owen et al., 2004). MMP‐8 is transcribed in PMN precursors in the bone marrow and preformed proMMP‐8 protein is stored within PMN secondary granules and is released from these granules when PMNs are activated. MMP‐8 is also expressed on the surface of activated PMNs where it contributes to pericellular proteolysis that is resistant to inhibition by physiologic inhibitors of soluble MMPs [tissue inhibitor of MMP (TIMPs) (Owen et al., 2004)]. Thus, the localization of MMP‐8 on the surface of PMNs fundamentally alters its biologic activities. TIMP‐1, an important inhibitor of soluble MMP‐8, is contained within the cytoplasmic vesicles of PMNs (Price et al., 2000). TIMP‐1 is released by degranulating PMNs and subsequently binds to the surface of PMNs where it serves as the receptor for active MMP‐8 on the surface of PMNs (Wang et al., 2019). Thus, surface‐bound TIMP‐1 on activated PMNs promotes pericellular proteolysis.

Macrophage‐derived MMPs make important contributions to the pathogenesis of chronic obstructive pulmonary disease (COPD), pulmonary fibrosis, acute lung injury syndromes, atherosclerosis, and aortic aneurysms (Craig et al., ,^2013^, ^2014^; Deguchi et al., 2005; Hergrueter et al., 2011; Herman et al., 2001; Owen, 2008b; Rabkin, 2017; Sethi et al., 2012). Macrophages synthesize MMP‐8 and TIMP‐1 de novo when activated (Herman et al., 2001). While activated PMNs release large quantities of MMP‐8 and relatively little TIMP‐1, activated monocytic cell lines (e.g., human THP‐1 cells) produce TIMP‐1 in excess of MMP‐8 (Opdenakker et al., 1991; Van Ranst et al., 1991). However, surprisingly little else is known about the cell biology of macrophage‐derived MMP‐8 and its contributions to physiologic and pathologic processes. Pro‐inflammatory stimuli such as CD40 ligand and TNF‐α induce the expression and release of MMP‐8 from monocyte‐derived macrophages (MDMs) in vitro (Herman et al. 2001). In addition, MMP‐8 is strongly up‐regulated in alveolar macrophages and peripheral blood monocytes from patients with idiopathic pulmonary fibrosis (Craig et al. 2014). MMP‐8 is also expressed by tissue macrophages in hyperoxia‐induced lung injury (Cederqvist et al. 2006), bronchiectasis (Prikk et al. 2001), and atherosclerotic plaques (Molloy et al. 2004).

Macrophages are thought to freely secrete all of the proMMP‐8 that they synthesize into the extracellular environment where proMMP‐8 is activated and then degrades extracellular proteins. However, it is not clear whether MMP‐8 and its major inhibitor in extracellular fluids (TIMP‐1) are expressed on the surface of activated macrophages and contribute to pericellular proteolysis that is associated with macrophages or their capacity to migrate through tissue barriers. Thus, we tested the hypotheses that: (1) MMP‐8 and TIMP‐1 are also expressed on the surface of activated macrophages, (2) surface‐bound MMP‐8 on macrophages promotes TIMP‐resistant pericellular proteolysis and macrophage migration through tissue barriers, and (3) MMP‐8 binds to TIMP‐1 on the surface of macrophages.

## 2 MATERIALS AND METHODS

### 2.1 Materials

*Escherichia coli* lipopolysaccharide (LPS) was obtained from Calbiochem. Molecular Probes Inc. (Eugene, OR) supplied goat anti‐rabbit Fab_2_‐Alexa Fluor 488^®^. Human proMMP‐8 and polyclonal rabbit anti‐human MMP‐8 IgG (AB8115) were purchased from Chemicon International Inc. R&D Systems provided murine anti‐MMP‐8 IgG (#908). Polyclonal rabbit anti‐human and anti‐murine MMP‐8 IgG (ab53017) was purchased from Abcam. Invitrogen (Carlsbad, CA) provided monoclonal mouse anti‐murine Timp‐1 IgG (F31 P2 A5 clone). DQ™‐quenched FITC‐conjugated type I collagen (acid‐solubilized) from bovine skin (catalog number D12060) was obtained from ThermoFisher Scientific. Biocoat™ Growth Factor Reduced Matrigel^™^ Invasion Chambers (catalog number 354483) were obtained from Corning Life Sciences. Type I collagen (acid‐solubilized) gel (PureCol^™^, catalog # 5005) was purchased from Advanced BioMatrix, Inc.). CalBiochem Novabiochem Corporation supplied (7‐Methoxycoumarin‐4‐yl)‐Acetyl‐Pro‐Leu‐Gly‐Leu‐(3‐[2,4‐dinitrophenyl]‐L2,3‐diamino‐propionyl)‐Ala‐Arg‐NH_2_ (McaPLGLDpaAR). All other reagents were purchased from Sigma‐Aldrich.

### 2.2 Ethics

#### 2.2.1 Human subjects

All studies performed on human subjects were approved by the Brigham and Women's Hospital Institutional Review Board, and all participants signed informed consent forms.

#### 2.2.2 Mice

All procedures conducted on mice were approved by the Harvard Medical School and Brigham and Women's Hospital Institutional Animal Care and Use Committees. *Mmp8* ^−/−^ mice [generated as described previously (Balbin et al., 2003)] were backcrossed to a pure C57BL/6 J strain (F10 generation). Parental littermate C57BL/6 J WT mice were studied as a control. *Timp*‐*1* ^−/−^ mice in a pure C67BL/6 J background were obtained from Paul Soloway (Cornell University). The *Timp*‐*1* ^−/−^ mice that we studied have been shown to lack Timp‐1 mRNA transcripts and/or protein in their leukocytes and other cells (Kim et al., 2001; Lee et al., 2005; Mohammed et al., 2005; Wang et al. & Owen, 2019). Age‐matched male and female mice were studied in all experiments.

### 2.3 Methods

#### 2.3.1 Human monocyte isolation and culture

Monocytes were isolated from blood samples obtained from healthy volunteers. Monocytes were isolated from blood samples using density gradient centrifugation with LSM^®^ Lymphocyte Separation Media (ICN Biomedical Inc. Aurora, OH), and subsequent adherence of the monocytes to plastic dishes or fibronectin‐coated glass cover slips (BioWhittaker Walkersville MD), a technique that yields monocyte preparations that are >92% pure (Owen et al., 1992, 1994). Monocyte‐derived macrophages (MDM) were differentiated in culture in a RPMI medium containing 2% human serum (Sigma Aldrich) for 10 days, and then the cells were stimulated at 37°C for varying times with 20 ng/mL LPS.

#### 2.3.2 Isolation and activation of murine peritoneal macrophages

Macrophages were isolated from the peritoneal cavities of WT, *Mmp*‐*8* ^−/−^, or *Timp*‐*1* ^−/−^ mice by performing peritoneal lavage 4 days after delivering 1 mL of a 4% solution of thioglycollate in Dulbecco's Modified Eagle Medium (DMEM) via the intra‐peritoneal route. Differential leukocyte counts performed on cytocentrifuge preparations showed that greater than 90% of the cells were macrophages. Macrophages were counted using a Coulter counter or a hemocytometer. Macrophages were allowed to adhere to 8‐well chamber slides (Nunc Inc.) and were activated with LPS (10 µg/mL), murine cluster of differentiation 40 (CD40) ligand (5 μg/mL), murine tumor necrosis factor‐α (Tnf‐α, 100 U/mL), or murine Ccl‐2 (10^−8 ^M) for 18 h at 37°C in a humidified atmosphere of 5% CO_2_. Cells were fixed with PBS containing 3% paraformaldehyde and 0.25% glutaraldehyde at 4°C for 5 min and then immunostained for surface‐bound Mmp‐8 as outlined below. Pilot dose optimization experiments were conducted, and the concentration of the agonist that induced maximal MMP‐8 surface MMP‐8 levels was selected for the results shown in this manuscript.

#### 2.3.3 Immunofluorescence staining of human MDM and murine macrophages and quantitative image analysis, and confocal microscopy

Cells were activated as outlined above and then fixed in PBS containing 3% paraformaldehyde and 0.25% glutaraldehyde (pH 7.4) for 3 min at 4°C. Cells were washed and then incubated for 1 h at 4°C in PBS containing 1% human serum albumin and 50 µg/mL goat IgG to reduce non‐specific binding of antibodies to cells. Polyclonal rabbit anti‐MMP‐8 IgG or non‐immune rabbit IgG was applied, followed by goat anti‐rabbit F(ab)_2_ conjugated to Alexa 488^®^. Cells were examined using epi‐fluorescence microscopy, images of the cells were acquired, and cell surface immunofluorescence was quantified using the MetaMorph® software as described previously (Owen et al., 1995). Images of immunostained cells were also acquired using a BioRad scanning confocal microscope equipped with a krypton–argon laser, and images were processed with a BioRad confocal assistant and Adobe Photoshop 7.0.

#### 2.3.4 Analysis of MDM and macrophage surface‐bound MMP‐8

The association of MMP‐8 with MDM membranes was assessed using surface labeling with biotin, purification of biotinylated surface proteins with immobilized avidin, and western blotting with a specific anti‐MMP‐8 antibody. LPS‐activated MDM were incubated with sulfo‐NHS‐biotin in PBS (pH 8.0) (Pierce) on ice for 20 min and washed with ice cold PBS, and 0.1 M glycine in PBS (pH 8.0) was applied. The cells were lyzed in radio‐immunoprecipitation buffer containing proteinase inhibitors (Sigma Aldrich). Aliquots of lysates and supernatants were reserved for analysis. Immunopure immobilized avidin gel (ThermoFisher Scientific) was prepared following the manufacturer's instructions. Biotinylated protein bound to avidin was dissociated by boiling the samples in SDS‐containing reducing buffer. Proteins were separated using sodium dodecyl sulfate polyacrylamide gel electrophoresis (SDS‐PAGE) and transferred to polyvinylidene difluoride membranes, and then probed with murine anti‐MMP‐8 IgG (diluted 1:1000) and goat anti‐murine IgG conjugated to horse radish peroxidase. Proteins were visualized using enhanced chemiluminescence (PerkinElmer, Boston, MA) (Herman et al. & Schonbeck 2001).

#### 2.3.5 MMP‐8 catalytic activity on the surface of human MDM and murine macrophages

Cells were activated and fixed as above, washed, and then resuspended in Tris assay buffer (0.05 M Tris containing 0.15 M NaCl and 0.02 M CaCl_2_; pH 7.4). This technique prevents cellular release of MMP‐8 and preserves the activity of membrane‐bound MMP‐8 on PMNs (Owen et al., 2004). To quantify cell surface type I collagenase activity associated with LPS‐activated human MDM or LPS‐activated murine peritoneal macrophages, equal numbers of cells were fixed, washed, and then pre‐incubated in duplicate for 2 h at 37°C with 1 mM phenylmethylsulfonyl fluoride (PMSF), 100 µM pepstatin, and 100 µM leupeptin to inactivate serine, aspartic acid, and cysteine proteinases, respectively. Equal numbers of cells (4 × 10^5^ cells/assay for macrophages and 9 × 10^5^ cells/assay for MDMs) were incubated for 24 h at 37°C with 50 µg/mL DQ™‐quenched FITC‐conjugated type I collagen (acid‐solubilized), and MMP‐mediated type I collagenase activity was quantified in cell‐free supernatants using fluorimetry (F2500 fluorescence spectrophotometer, Hitachi Ltd. Tokyo; and λ_ex_ 490 nm, λ_em_ 520 nm). Substrate cleavage was quantified as arbitrary fluorescence units.

#### 2.3.6 Type I collagenase activity of exogenous active Mmp‐8 bound to the surface of murine macrophages and its susceptibility to inhibition by Timp‐1

Equal numbers of bone marrow‐derived macrophages from WT mice were incubated at 4°C with: (1) no exogenous Mmp‐8 for 2 h, (2) 200 nM amino‐phenyl mercuric acetate (APMA)‐activated purified murine Mmp‐8 for 2 h, (3) 200 nM APMA‐activated purified murine Mmp‐8 for 1 h followed by 400 nM Timp‐1 for 1 h, and (4) 200 nM APMA‐activated purified Mmp‐8 for 1 h and then 20 µM GM6001 (a low molecular weight Mmp inhibitor) as described previously (Wang et al., 2019). The cells were fixed and washed with buffer three times and then incubated (12 × 10^6^ cells/assay) at 37°C with type‐I collagen conjugated to DQ™‐quenched‐FITC for 18 h. Mmp‐8‐mediated cleavage of the substrate was quantified in cell‐free supernatant samples using fluorimetry as described above. The data were expressed as a % cleavage of the substrate in cells incubated with active Mmp‐8 without inhibitor (n = 4 separate experiments).

#### 2.3.7 Macrophage migration through artificial matrix and smooth muscle cell (SMC) layers

Migration studies were conducted using 8 µM pore Biocoat™ Growth Factor Reduced Matrigel^™^ Invasion Chambers (according to the manufacturer's instructions) or Falcon™ 24‐well cell culture inserts (Becton Dickenson). The upper chamber inserts were coated with 50 µL of acid‐solubilized type‐I collagen gel (PureCol^™^, Advanced BioMatrix, Inc.). Layers of murine SMC (isolated as described previously) and their associated matrix were generated by growing SMC for 5–7 days on gelatin‐coated inserts in DMEM containing 20% fetal calf serum (Gerdes et al., 2002). Macrophages (3 × 10^5^/mL) were seeded into the inserts in serum‐free RPMI medium. Chemoattractants studied in the lower chamber were 10% serum or 30 nM recombinant murine Ccl‐2 (BD Biosciences). The cells were incubated at 37°C for 24 h and then stained in modified Wright's stain. The mean number of cells per microscopic field was counted on triplicate filters for each experimental condition. All experiments were repeated three times.

#### 2.3.8 Recruitment of macrophages during experimental acute peritonitis

Acute sterile peritonitis was induced in age‐ and sex‐matched WT and *Mmp*‐*8* ^−/−^ mice using thioglycollate, as described above. Peritoneal lavage was performed using 3 × 10 mL aliquots of RPMI medium, and erythrocytes were removed using hypotonic lysis. The number of peritoneal leukocytes per mouse was counted using a hemocytometer, and the percentage of macrophages in the samples was assessed by differential cell counts performed on modified Wright's stained cytocentrifuge preparations. The number of macrophages isolated per mouse was calculated.

#### 2.3.9 Quantification of surface Timp‐1 or Mmp‐8 on murine macrophages

Macrophages were isolated from the peritoneal cavities of WT or *Timp*‐*1* ^−/−^ mice, as described above. Macrophages were rendered quiescent by seeding them at 2 × 10^5^ cells per well in chamber slides in DMEM containing 5% fetal bovine serum, supplemented with penicillin (100 U/mL), streptomycin (100 μg/mL), and amphotericin B (0.25 μg/mL) for 4 days at 37°C in a humidified atmosphere of 5% CO_2_. The cells were then incubated with or without LPS (10 μg/mL) for 18 h at 37°C. WT macrophages were fixed (as described above) and then stained for cell surface‐bound Timp‐1. Cells were incubated overnight at 4°C in PBS containing 1% albumin and 50 µg/mL goat IgG to block non‐specific binding of antibodies to the cells. Cells were immunostained with murine anti‐murine Timp‐1 IgG (MA1‐773) or non‐immune murine IgG applied at the same concentration for 18 h at 4°C, followed by goat‐anti‐murine F(ab)_2_ conjugated to Alexa‐488 at 37°C for 2 h.

In other experiments, unstimulated and LPS‐activated WT and *Timp*‐*1* ^−/−^ macrophages were fixed and then immunostained for surface‐bound Mmp‐8. Cells were incubated overnight at 4°C in PBS containing 1% albumin and 5% normal goat serum. Cells were then incubated with rabbit anti‐murine Mmp‐8 IgG (ab53017) or non‐immune rabbit IgG applied at the same concentration for 18 h at 4°C, followed by goat‐anti‐rabbit F(ab)_2_ conjugated to Alexa‐488 at 37°C for 2 h.

Nuclei were counterstained with 4′,6‐diamidino‐2‐phenylindole (DAPI) mounting gel (Abcam, Cambridge, MA). Cells were examined with a Leica epi‐fluorescence microscopy, and images of the stained cells were acquired. Cell surface immunofluorescence was quantified using the image analysis software (MetaMorph™ software, Universal Imaging Inc., West Chester, PA), and the data were corrected for non‐specific staining, as described previously (Owen et al., 1995).

#### 2.3.10 Statistics

Data are presented as mean + SEM (if normally distributed), or as box plots showing medians and 25th and 75th percentiles, and whiskers showing 10th and 90th percentiles (if not normally distributed). The results for paired and unpaired data were compared using Student's t‐test for parametric data and the Mann–Whitney rank sum test for non‐parametric data; *p* values less than 0.05 are significant.

## 3 RESULTS

### 3.1 MMP‐8 is expressed on the surface of activated human MDM and murine macrophages

**FIGURE 1:**
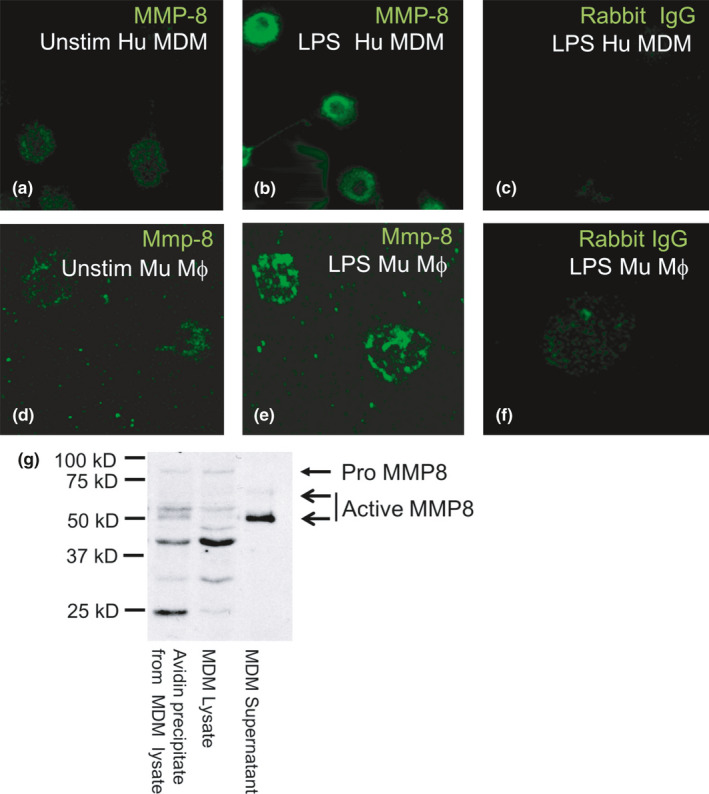
MMP‐8 is localized on the surface of mononuclear phagocytes, as assessed by confocal microscopy and biotinylation of surface proteins. Confocal micrographs show human (Hu) monocyte‐derived macrophages (MDM; a–c) and murine macrophages (Mu Mϕ; d–f) grown on coverslips that were either not stimulated (Unstim) or incubated for 24 h with bacterial lipopolysaccharide (LPS) and then fixed (but not permeabilized) and immunostained with a green fluorophore for surface MMP‐8. There was low‐level MMP‐8 staining on the surface of unstimulated human MDM and murine macrophages (a and d) and increased staining for MMP‐8 on LPS‐activated cells (b and e). Cells incubated with the isotype‐matched control antibody show minimal non‐specific staining (c and f). (g) Proteins on the surface of human MDM or proteins released by the cells were labeled with biotin. Unbound biotin was quenched with glycine, and cells and cell‐free supernatant fluids were separated by centrifugation. Cells were lyzed, and surface biotinylated proteins were captured using immobilized avidin, separated by SDS‐PAGE, and then immunoblotted with a murine monoclonal anti‐MMP‐8 antibody, which recognizes pro‐, active, and further processed MMP‐8. Biotinylated surface proteins of LPS‐stimulated macrophages avidin‐precipitated from cell lysates (lane 1, 50 µL) were compared to total cell lysate (removed before the avidin precipitation step; lane 2, 50 µL) and cell supernatant that was not subjected to avidin precipitation (lane 3, 50 µL) by western blotting with a monoclonal mouse anti‐MMP‐8 antibody. Bands corresponding to pro and active MMP‐8 are indicated by the arrows. Note that surface biotinylation of MDM demonstrates that most of the MMP‐8 forms that are present in macrophage lysates are also detected on the surface of the cells. The results shown are representative of 3 separate experiments

Non‐permeabilized unstimulated versus LPS‐activated human MDM (Figure 1a‐c) and murine macrophages (Figure 1d‐f) were fixed and then immunostained for cell surface‐bound MMP‐8. Analysis with a confocal microscope localized MMP‐8 to the surface of the LPS‐activated cells. Unstimulated human and murine macrophages had minimal quantities of MMP‐8 on their surface (Figure 1a,d), but LPS‐stimulated cells displayed intense focal cell surface staining for MMP‐8 (Figure 1b,e). Cells immunostained with a non‐immune primary antibody showed little or no staining (Figure 1c,f).

Biotin labeling of proteins on the surface of human MDM, avidin precipitation of surface biotinylated proteins, and subsequent immunoblotting for MMP‐8 identified several forms of immuno‐reactive MMP‐8 on the surface of MDMs (Figure 1g). The avidin‐complexed fraction represented biotinylated cell surface MMP‐8 forms (lane 1), while an aliquot of the same lysate that was not subjected to avidin precipitation reflected intracellular and surface MMP‐8 forms (lane 2). The supernatant sample (lane 3) contained MMP‐8 that was freely released by the cells in response to LPS stimulation (Figure 1g). ProMMP‐8 was present both in the total cell lysate and the biotinylated fraction, verifying the presence of proMMP‐8 on the cell surface. The supernatant contains a 64 kD form of MMP‐8 corresponding to the active form, as described previously (Herman et al., 2001). The cell surface, supernatant, and total cell lysates also contained 50 and 55 kDa forms similar to the catalytically active non‐glycosylated forms of MMP‐8 that are produced by endothelial cells and fibroblasts (Hanemaaijer et al., 1997). In addition, there were three lower M_r_ bands (24–40 kDa) which have been reported previously for MMP‐8 (Knauper et al., 1997) including MMP‐8 in lysates of specific granules isolated from PMNs and MMP‐8 present in the plasma membrane fraction of activated PMNs (Owen et al., 2004).

### 3.2 Pro‐inflammatory mediators increase surface MMP‐8 protein levels on human MDM and murine macrophages

**FIGURE 2:**
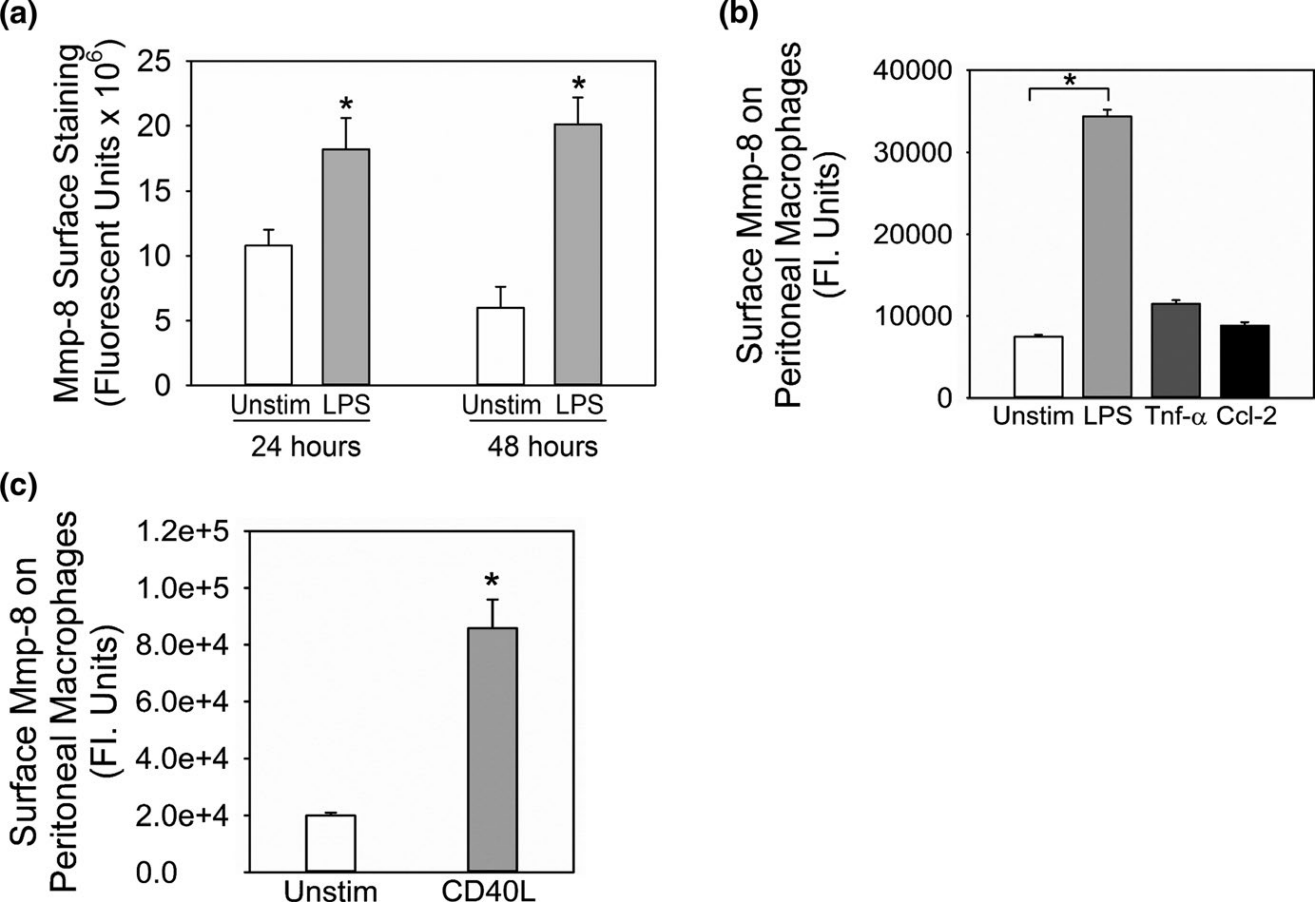
LPS and cluster of differentiation 40 ligand increase the expression of MMP‐8 on the surface of mononuclear phagocytes. In (a) human monocyte‐derived macrophages (MDM) from 4 different donors were cultured on coverslips, and incubated without (Unstim) or with 20 ng/mL bacterial lipopolysaccharide (LPS) for 24 and 48 h. Cells were fixed, and surface MMP‐8 was detected using immunostaining and quantified using image analysis software. Results are corrected for non‐specific staining using macrophages stained with an isotype‐matched control primary antibody. Data shown are mean +SEM; n = 67–91 cells/group. Asterisk indicates *p* = 0.033 at 24 h and *p* < 0.001 at 48 h versus the unstimulated group. In B‐C, macrophages were isolated from the peritoneal cavities of WT mice with thioglycollate‐induced sterile peritonitis. Cells were cultured in chamber slides in medium without agonists for 48 h to render the cells quiescent. The cells were then incubated with or without 10 μg/mL LPS, 100 U/mL murine tumor necrosis factor‐ α (Tnf‐α), or 10^−8 ^M murine Ccl‐2 (B), or murine cluster of differentiation 40 ligand (CD40L; C) for 24 h at 37°C. Cells were then fixed and immunostained for surface Mmp‐8 which was quantified using image analysis software. Data are mean + SEM; n = 500 cells/group. The result shown is representative of 3 separate experiments. In B, asterisk indicates *p* < 0.01 versus unstimulated cells, and in C, *indicates *p* < 0.001 versus unstimulated cells at the same time point

Quantitative analysis of unstimulated versus LPS‐activated human MDM that were immunostained for MMP‐8 revealed the modest constitutive expression of MMP‐8 on the surface of unstimulated MDM (Figure 2a). Stimulating the cells for 24 h with LPS increased surface MMP‐8 staining by 1.5‐ to 3‐fold. The LPS‐induced increase in MMP‐8 surface staining on human MDM was detected after 6 h (*data not shown*), peaked at 24 h, and remained elevated at 48 h (Figure 2a). Activation of WT macrophages with LPS (Figure 2b) and murine CD40 ligand (Figure 2c) resulted in 3‐ and 5‐fold increases, respectively, in surface Mmp‐8 staining compared with unstimulated cells. Neither murine Tnf‐α nor murine Ccl‐2 [a potent chemokine for monocytes (Owen et al., 1994)] increased murine macrophage surface Mmp‐8 levels (Figure 2b).

### 3.3 Surface MMP‐8 on murine macrophages and human MDMs is catalytically active

**FIGURE 3:**
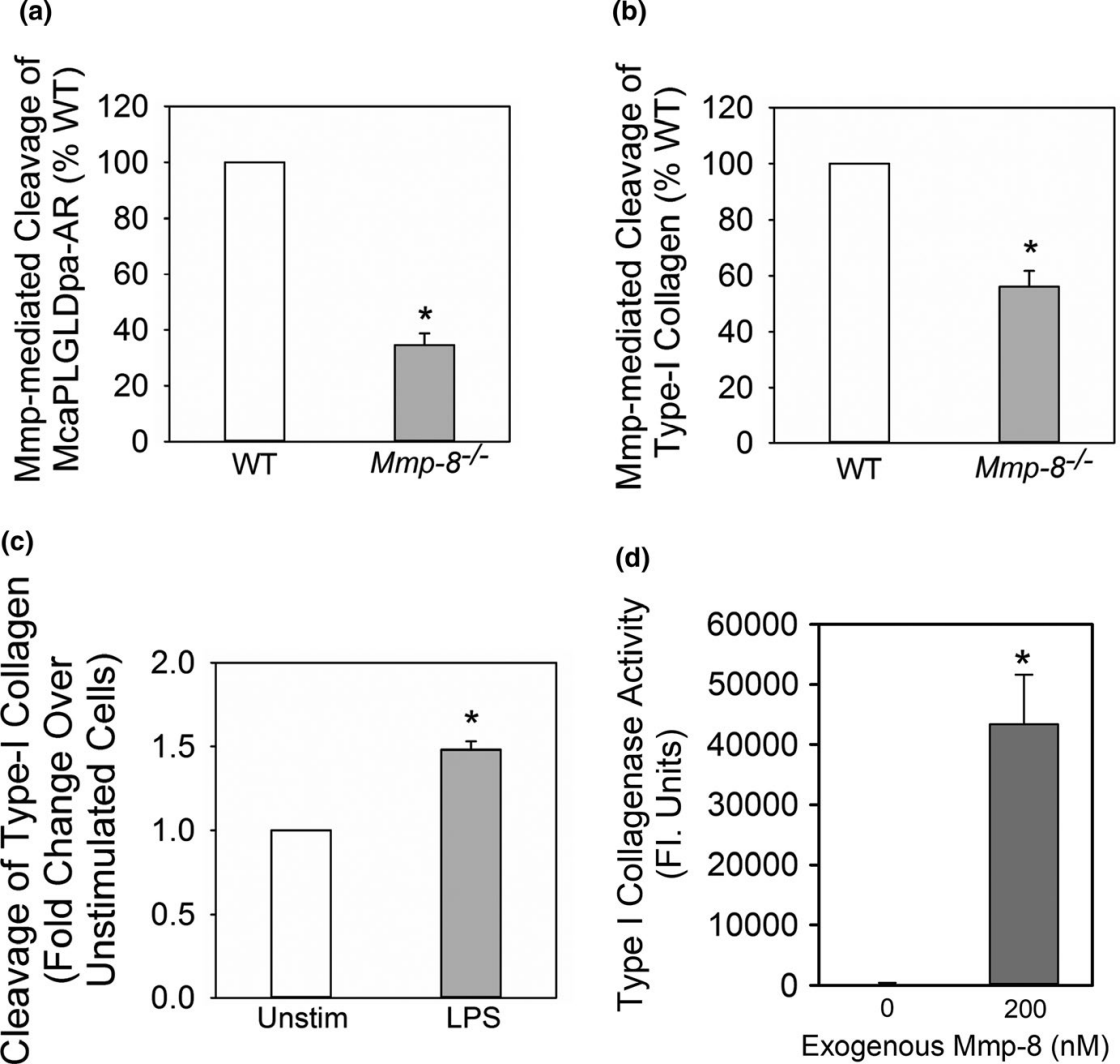
Membrane‐bound Mmp‐8 on macrophages is catalytically active and degrades type I collagen: In (a and b), equal numbers of bacterial lipopolysaccharide (LPS)‐activated wild‐type (WT) and *Mmp‐8* ^−/−^ macrophages were incubated with McaPLGLDpaAR (a quenched fluorescent peptide substrate which is robustly cleaved by Mmps) in A or FITC‐conjugate type I collagen in B in the presence and absence of 1,10‐o‐phenanthroline (a non‐selective inhibitor of metalloproteinases). Mmp‐mediated (1,10‐o‐phenanthroline sensitive) cleavage of the substrate was quantified using fluorimetry. Data are mean ± SEM; n = 3 cell preparations. Asterisk indicates *p* < 0.01. (c) LPS‐activated human monocyte‐derived macrophages were fixed (to prevent release of soluble Mmp‐8) and then incubated with quenched fluorescent type I collagen as a substrate. MMP‐mediated (1,10‐phenanthroline‐sensitive) type I collagenase activity associated with the cell surface was quantified using a fluorimeter. Data are mean ±SEM; n = 3 separate experiments using cells isolated from 3 different donors were used. Asterisk indicates *p* = 0.029. (d) Equal numbers of bone marrow‐derived macrophages from WT mice were incubated at 4°C with or without 200 nM amino‐phenyl mercuric acetate (APMA)‐activated purified murine Mmp‐8 for 2 h. The cells were fixed and washed with buffer three times and then incubated at 37°C with type I collagen conjugated to quenched FITC for 18 h. Mmp‐8‐mediated cleavage of substrate was quantified in arbitrary fluorescence units in cell‐free supernatant samples using fluorimetry, as described in Methods. Data are mean + SEM; n = 4 separate experiments

We compared the activity of Mmp‐8 on the surface of fixed, LPS‐activated WT and *Mmp*‐*8* ^−/−^ macrophages against two quenched fluorogenic substrates that generate a fluorescent signal only when cleaved: (1) a peptide substrate (McaPLGLDpaAR), which is sensitive to cleavage by most Mmps (Figure 3a), and (2) DQ™‐quenched‐FITC‐conjugated type I collagen, which is a sensitive substrate for Mmp‐8 (Figure 3b). *Mmp*‐*8* ^−/−^ macrophages had 65% less activity against McaPLGLDpaAR and 45% less type I collagenase activity than that associated with WT cells indicating that surface Mmp‐8 on LPS‐activated murine macrophages contributes 65% of the total cell surface Mmp activity and 45% of the surface type I collagenase activity associated with activated WT murine macrophages (Figure 3a,b). The increase in MMP‐mediated type I collagenase activity on the surface of LPS‐activated human MDMs (Figure 3c) was similar in magnitude to the increase in immuno‐reactive MMP‐8 detected on LPS‐activated cells (Figure 2a).

To provide further assurance that active Mmp‐8 bound to the surface of murine macrophages is catalytically active, we incubated equal numbers of unstimulated WT macrophages at 4°C with and without exogenous active Mmp‐8 to permit Mmp‐8 to bind to the cell surface. Cells were washed to remove unbound Mmp‐8 and fixed, and then the surface‐associated type I collagenase activity was measured. There was no measurable type I collagen‐degrading activity associated with WT macrophages that had not been incubated with Mmp‐8, but robust cleavage of this substrate was associated with cells that had bound Mmp‐8 to their surface (Figure 3d).

### 3.4 Surface Mmp‐8 activity on murine macrophages is resistant to inhibition by Timps

**FIGURE 4:**
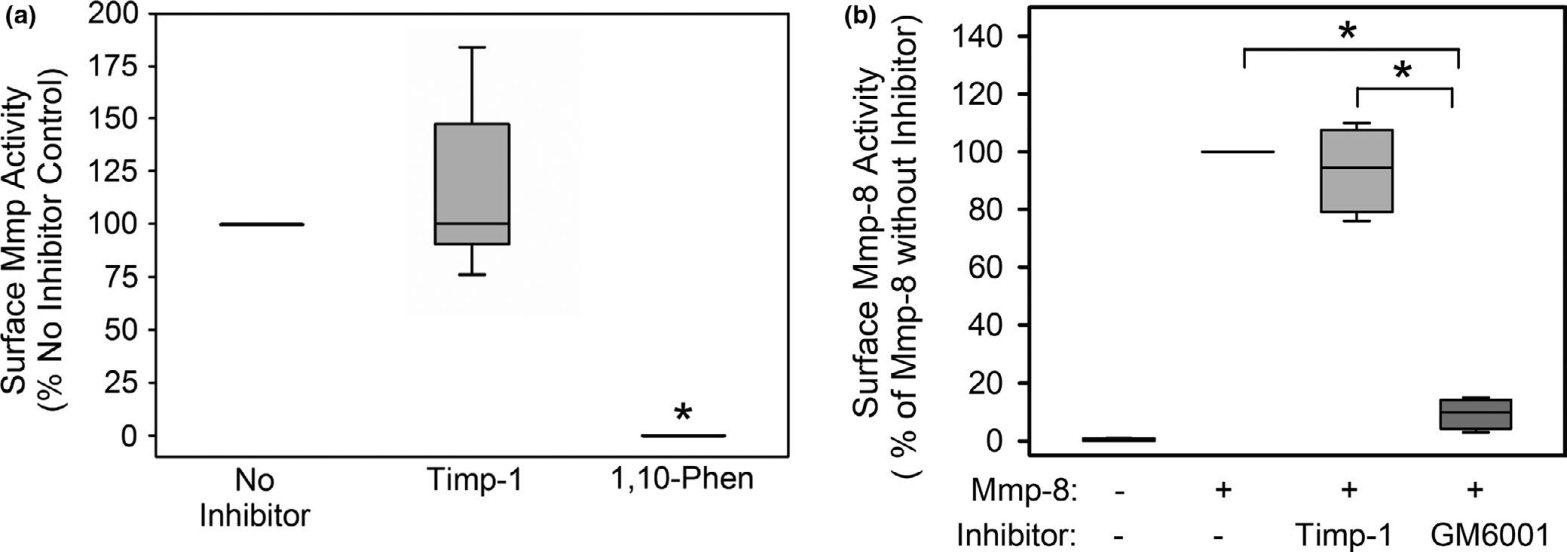
Membrane‐bound Mmp‐8 on murine macrophages is resistant to inhibition by Timps but not a low molecular weight synthetic Mmp inhibitor: In A, activated WT peritoneal macrophages were fixed, and then incubated with 500 nM Timp‐1 (n = 7 cell preparations) or 1 mM 1,10‐o‐phenanthroline (1,10‐phen; a low molecular weight non‐selective metalloproteinase inhibitor; n = 3 cell preparations) or no inhibitor (n = 7 preparations, as a control) for 2 h at 37°C. Residual surface Mmp activity was measured using a sensitive, quenched, fluorogenic peptide substrate for Mmps (McaPLGLDpaAR) for 4 h at 37°C and cleavage of this substrate was quantified using fluorimetry. The data are expressed as a % of the no inhibitor control. The boxes in the box‐plots show the medians and 25^th^ and 75^th^ percentiles and the whiskers show the 10^th^ and 90^th^ percentiles. Asterisk indicates *p* < 0.05 versus the no inhibitor control. In B, equal numbers of bone marrow‐derived macrophages from WT mice were incubated at 4°C with: (1) no exogenous murine Mmp‐8 for 2 h; (2) 200 nM amino‐phenyl mercuric acetate (APMA)‐activated purified murine Mmp‐8 for 2 h; (3) 200 nM APMA‐activated purified murine Mmp‐8 for 1 h followed by 400 nM murine Timp‐1 for 1 h; (4) 200 nM APMA‐activated purified Mmp‐8 for 1 h and then 20 µM GM6001 (a low molecular weight non‐selective metalloproteinase inhibitor; M_r_ 388 Da). The cells were fixed and washed with buffer three times and then incubated at 37°C with type I collagen conjugated to quenched FITC for 18 h. Mmp‐8‐mediated cleavage of substrate was quantified in cell‐free supernatant samples using fluorimetry, as described in Methods. The data are expressed as a % cleavage of the substrate in cells incubated with active Mmp‐8 without inhibitor (n = 4 separate experiments). The boxes in the box‐plots show the medians and 25^th^ and 75^th^ percentiles and the whiskers show the 10^th^ and 90^th^ percentiles. Data were analyzed using a Kruskal‐Wallis One‐Way ANOVA followed by pair‐wise testing with Mann–Whitney U tests. Asterisk indicates *p* < 0.05 versus the group indicated

To determine whether the surface Mmp‐8 activity associated with LPS‐activated murine macrophages is susceptible to inhibition by Timps, activated WT peritoneal macrophages were incubated with or without 500 nM murine Timp‐1 or 1,10‐o‐phenanthroline, a low molecular weight inhibitor of Mmps for 2 h, and then the residual surface Mmp activity was measured using a sensitive peptide substrate for MMPs. Adding Timp‐1 to the cells resulted in no inhibition of surface Mmp activity, whereas 1,10‐o‐phenanthroline completely inhibited the surface Mmp activity that was associated with the activated macrophages (Figure 4a).

Additional experiments were performed to provide further assurance that among all of the metalloproteinases expressed on the surface of LPS‐activated macrophages, surface‐bound Mmp‐8 is resistant to inhibition by Timp‐1. Unstimulated WT macrophages were incubated with exogenous active murine Mmp‐8 at 4°C to permit binding of the Mmp‐8 to the surface of the cells. The cells were then incubated with or without murine Timp‐1 or GM6001 (a small molecular weight non‐selective inhibitor of metalloproteinases; M_r_ 388 Da). The cells were then washed and fixed, and the cell surface‐associated type I collagen degrading activity was measured using fluorimetry. Exogenous Timp‐1 did not inhibit the type I collagen‐degrading activity associated with exogenous active Mmp‐8 bound to the surface of WT macrophages, whereas GM6001 was 100% effective at inhibiting this activity (Figure 4b).

### 3.5 Activities of Mmp‐8 in macrophage migration

**FIGURE 5:**
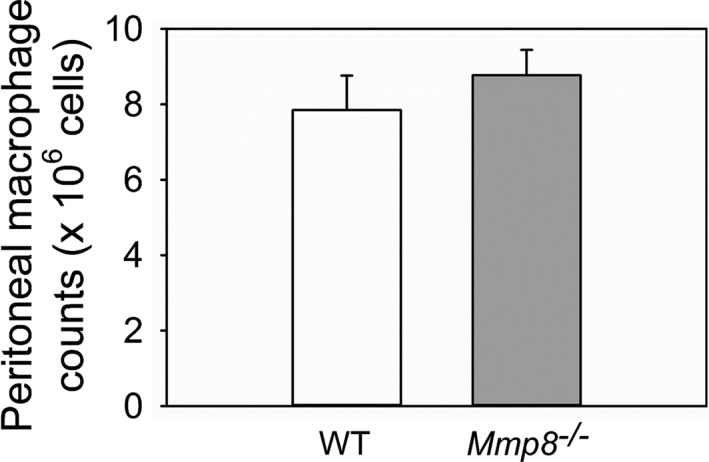
Mmp‐8 is not essential for the accumulation of macrophages in the peritoneal cavities of mice during sterile acute peritonitis: Wild‐type (WT) and *Mmp*‐*8* ^−/−^ mice were treated with a single injection of thioglycollate solution via the intraperitoneal route. Four days later, peritoneal lavage was performed, and peritoneal macrophages were counted. Data are mean + SEM; n = 8 mice/group; *p* = 0.417

We determined whether active Mmp‐8 expressed on the surface of activated monocytes and/or macrophages contributes to the capacity of monocytes/macrophages to migrate including into a serosal sac (the peritoneum), which is thought to be dependent on proteinase‐independent amoeboid‐type macrophage migration (Verollet et al., 2015). The accumulation of macrophages in the peritoneal cavities of mice during thioglycollate‐induced peritonitis was compared in WT and *Mmp*‐*8* ^−/−^ mice. WT and *Mmp*‐*8* ^−/−^ mice with acute peritonitis had similar numbers of macrophages accumulating in their peritoneal cavities indicating that Mmp‐8 is not essential for macrophages to accumulate in a serosal sac, as expected (Figure 5).

**FIGURE 6:**
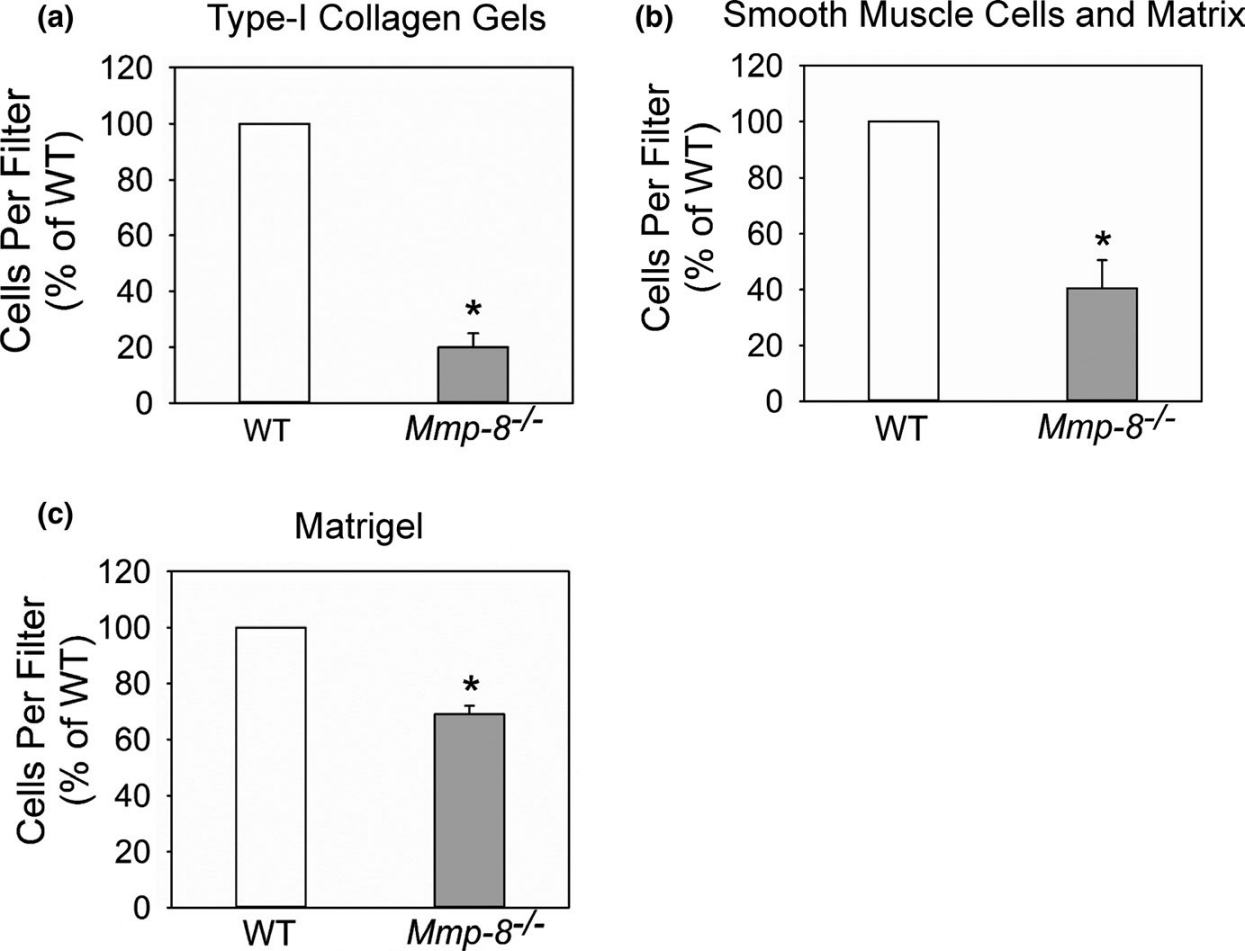
Mmp‐8 contributes to macrophage migration through collagen and Matrigel, and smooth muscle cell monolayers. In (a), the migration of equal numbers of wild‐type (WT) and Mmp8^−/−^ peritoneal macrophages placed in the upper chambers though a cell culture insert containing a type I collagen gel on top of an 8 µm pore membrane in response to 10% serum in the lower wells was quantified. In (b), the migration of equal numbers of WT and *Mmp‐8* ^−/−^ peritoneal macrophages placed in the upper chambers though a cell culture insert on which a confluent layer of murine smooth muscle cells were grown on an 8 µm pore membrane. Murine CC chemokine ligand‐2 (Ccl‐2; 2 nM) was added to the lower wells (as a chemoattractant for macrophages). In (c), the migration of equal numbers of WT and *Mmp* *‐* *8* ^−/−^ peritoneal macrophages placed in the upper chambers though a cell culture insert containing Matrigel™ on top of an 8 µm pore membrane in response to 10% serum in the lower wells was quantified. In A‐C, results are normalized to the value for WT cells. Experiments were carried out in triplicate with triplicate inserts per condition in each experiment. Data are mean + SEM. Asterisk indicates *p* ≤ 0.003 versus WT cells

Monocytes and macrophages can also migrate using the proteinase‐dependent mesenchymal mode involving podosomes (adhesive cellular structures) and proteinases to create paths through dense and poorly porous matrices (Guiet et al., 2012). As MMP‐8 expressed on the surface of activated mononuclear phagocytes contributes to pericellular proteolysis (Figures 1, 2, 3, 4), we investigated whether Mmp‐8 contributes to the migration of murine macrophages and their penetration of in vitro models of tissue barriers that vary in their density and content of type I collagen (and thus may require mesenchymal‐type macrophage migration). We hypothesized that macrophage migration through tissue barriers containing significant amounts of type I collagen (e.g., fibrotic organs) would be more dependent on the Mmp‐8 type I collagenase activity than that through an in vitro model of a vascular wall [smooth muscle cells (SMCs) and their accumulated ECM proteins] or Matrigel^™^ (which is predominantly composed of laminin, type IV collagen, and proteoglycans). As expected, *Mmp*‐*8* ^−/−^ macrophages had impaired migration through all barriers tested and their migration was impaired to a greater extent through type I collagen gels (by ~80%; Figure 6a) than through an in vitro model of a vascular wall (by ~60%; Figure 6b), or Matrigel™ (by ~33.3%; Figure 6c). Thus, Mmp‐8 contributes significantly to macrophage migration through type I collagen‐containing barriers.

### 3.6 Timp‐1 is expressed on the surface of activated murine macrophages but does not serve as the receptor to surface‐bound Mmp‐8

**FIGURE 7:**
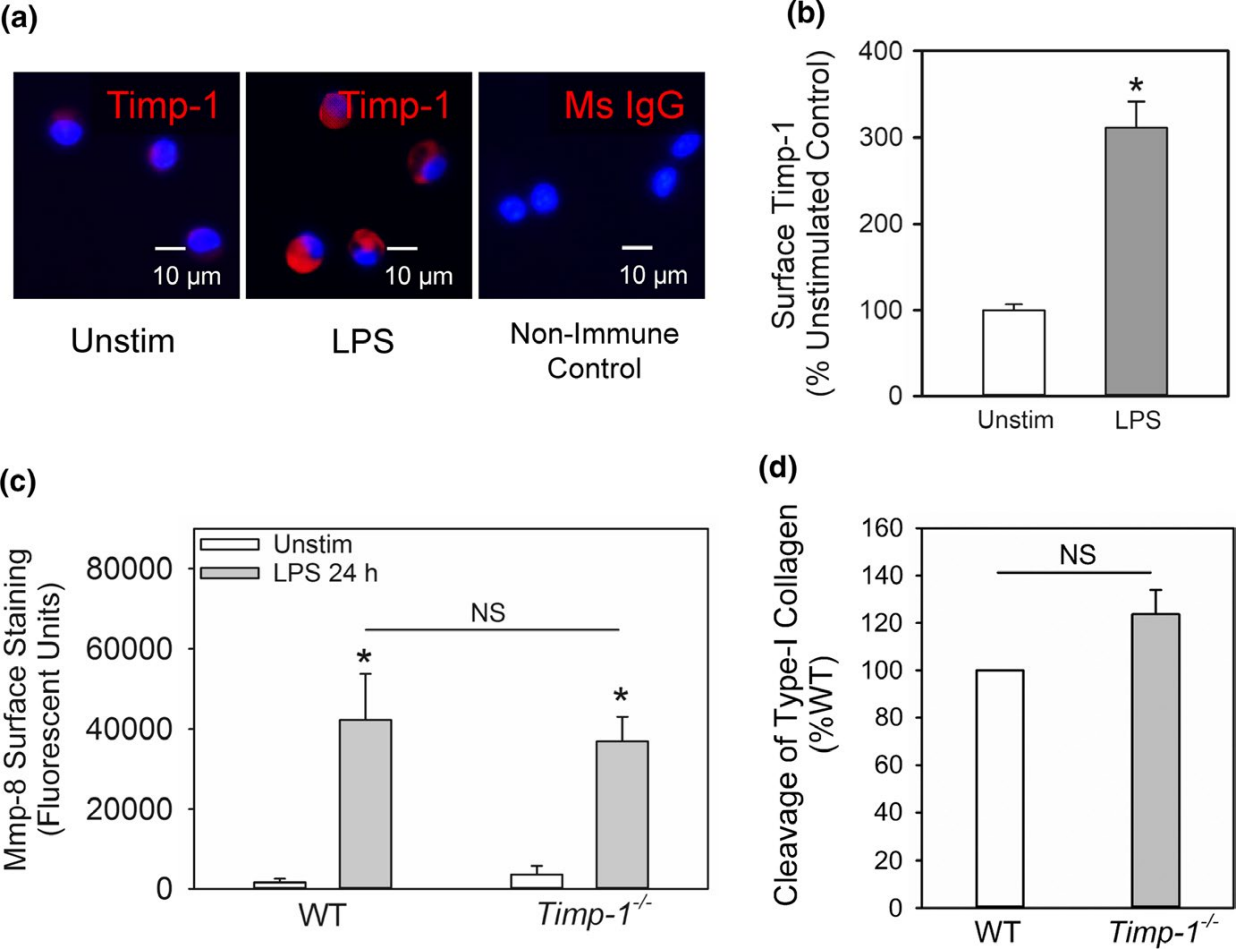
Timp‐1 is localized on the surface of activated macrophages, but does not serve as the receptor for Mmp‐8 on macrophages. Quiescent WT peritoneal macrophages were incubated without (Unstim) or with 10 µg/mL bacterial lipopolysaccharide (LPS) for 18 h, fixed, and then immunostained with a red fluorophore and an antibody to Timp‐1 or a non‐immune primary antibody (murine IgG; Ms IgG). Images shown in (a) are representative of 3 different cell preparations. In (b) surface Timp‐1 staining on unstimulated (Unstim) versus LPS‐activated wild‐type (WT) macrophages was quantified, as described in Methods. Data are mean + SEM; n = 500 cells in 3 separate experiments. In (c‐d), WT and *Timp‐1* ^−/−^ peritoneal macrophages were activated with LPS 10 µg/mL LPS for 18 h, fixed, and then either immunostained for surface‐bound Mmp‐8 (c) or surface type I collagenase activity was measured on equal numbers of cells (5 × 10^6^ cells/assay) using type I collagen conjugated to quenched FITC as the substrate and fluorimetry (d), as described in Methods. In (c‐d), data are mean + SEM; n = 3 different cell preparations

TIMP‐1 is expressed on the surface of activated human and murine PMNs where it serves as a receptor for active MMP‐8 (Wang et al., 2019). Thus, we investigated whether TIMP‐1 is also expressed in an inducible fashion on the surface of macrophages and anchors MMP‐8 to the surface of these cells. WT peritoneal macrophages were incubated with or without LPS for 18 h and immunostained for cell surface‐bound Timp‐1. There was minimal staining of Timp‐1 on the surface of unstimulated WT macrophages, but LPS induced a 3‐fold increase in surface Timp‐1 staining (Figure 7a,b). Surface Mmp‐8 levels were measured on LPS‐activated WT and *Timp*‐*1* ^−/−^ macrophages, as assessed by immunostaining the cells for Mmp‐8 and quantifying the surface type I collagenase activity associated with equal numbers of cells. LPS‐activated WT and *Timp*‐*1* ^−/−^ macrophages expressed similar quantities of surface‐bound Mmp‐8 as assessed by immunostaining (Figure 7c) and surface‐associated type I collagenase activity (Figure 7d) indicating that surface Timp‐1 is unlikely to be involved in anchoring Mmp‐8 to the surface of LPS‐activated macrophages.

## 4 DISCUSSION

Although MMP‐8 is known to be synthesized and freely secreted by activated macrophages (Herman et al., 2001), neither the key bioactive forms of macrophage‐derived MMP‐8 nor the contributions of MMP‐8 to macrophage function were investigated in previous studies. To the best of our knowledge, we report for the first time that MMP‐8 is expressed on the surface of activated human and murine mononuclear phagocytes and that surface‐bound MMP‐8 on macrophages is catalytically active and degrades type‐I collagen but is resistant to inhibition by TIMP‐1. Mmp‐8 is not required for murine monocytes to migrate into the peritoneal cavities of mice with acute peritonitis, which is consistent with the use of proteinase‐independent amoeboid migration of monocytes. However, Mmp‐8 is required for murine macrophages to migrate through in vitro models of dense tissue barriers especially those rich in type I collagen. The latter migratory mode is likely to be mediated by surface‐bound Mmp‐8 expressed on migrating murine macrophages via its degradation of type I collagen in the pericellular environment. In addition, Timp‐1 is expressed in an inducible fashion of the surface of murine macrophages. However, unlike surface‐bound Timp‐1 on murine PMNs (Wang et al., 2019), surface‐bound Timp‐1 on activated macrophages is unlikely to anchor Mmp‐8 to macrophage surfaces.

Although MMPs were initially thought to function as soluble proteinases, there is growing evidence that some MMPs lacking a transmembrane domain are localized on the surface of cells (other than monocytes and macrophages) where they contribute to pericellular proteolysis. To our knowledge, there have been no prior studies investigating whether macrophages express MMPs lacking a transmembrane domain or TIMPs on their surface. Until now, the only MMPs that were known to be expressed on monocyte and macrophage surfaces are members of the membrane‐type MMP (MT‐MMP) family (MT1‐MMP, MT2‐MMP, MT3‐MMP, and MT4‐MMP), which have an integral transmembrane domain that anchors them to cell surfaces (Owen, 2008a). MMPs lacking a transmembrane domain are expressed on the surface of other cells including MMP‐1 on keratinocytes (Dumin et al., 2001), MMP‐2 on the surface of endothelial cells, fibroblasts, and tumor cells (Brooks et al., 1996; Itoh et al., 2001; Will et al., 1996), and MMP‐8 and MMP‐9 on the surface of PMNs (Owen et al., 2003, 2004). In addition, MMP‐9 binds via its hemopexin domain to the α4β1 integrin on leukemia cells to promote cellular migration and survival (Redondo‐Munoz et al., 2008, 2010). We now report that MMP‐8 is localized on the surface of macrophages.

Biologically relevant mediators (bacterial LPS and CD40 ligand) increased surface levels of immunoreactive MMP‐8 on human MDM and/or murine macrophages in vitro, and these increases were associated with concomitant increases in the surface‐bound type I collagenase activity. Analysis of the forms of MMP‐8 present on the surface of MDM by western blotting revealed pro‐MMP‐8 forms having the same molecular mass as active MMP‐8 (~50–55 kDa) and forms having lower molecular mass. It is possible that the latter forms are likely generated by proteolytic processing of MMP‐8 including autocatalytic cleavage of MMP‐8 by the active site of MMP‐8 or via cleavage of MMP‐8 mediated by other MMPs expressed on macrophage surfaces (e.g., MT‐MMPs) or other MMPs secreted by macrophages including MMP‐9 and MMP‐12 (Owen & Campbell, 1999). How pro‐MMP‐8 is activated on the surface of MDM and macrophages is not clear. However, it is possible that oxidants and other proteinases released by MDM and macrophages participate in activation of latent proMMP‐8 before or after it binds to the cell surface (Owen & Campbell, 1999).

Studies of LPS‐activated WT and *Mmp*‐*8* ^−/−^ macrophages showed that Mmp‐8 accounts for a substantial proportion of the cell surface type I collagenase activity associated with activated macrophages. The cell surface type I collagenase activity expressed on LPS‐activated WT murine macrophages was resistant to inhibition by Timp‐1 but not a low molecular weight synthetic Mmp inhibitor in vitro. Studies of exogenous active Mmp‐8 bound to the surface of unstimulated WT macrophages confirmed that the surface‐bound Mmp‐8 activity on macrophages is resistant to inhibition by Timp‐1. These findings support the notion that surface‐bound Mmp‐8 on macrophages contributes to the macrophage pericellular collagenase activity in microenvironments *in vivo* in which there are high concentrations of extracellular proteinase inhibitors. We hypothesize that steric hindrance of Mmp‐8 that is spatially confined on a cell surface is likely responsible for its resistance to inhibition by Timps but not by low molecular weight synthetic Mmp inhibitors (Owen et al., 2004). The localization of Mmp‐8 on the macrophage surface not only shelters it from Timp inhibition but may also concentrate its activity on the surface to restrict Mmp‐8‐mediated proteolysis to the pericellular environment and prevent tissue injury.

Until now, the function of MMP‐8 that is expressed by macrophages and its contributions to macrophage migration have not been clear. Leukocytes migrate through tissues in three‐dimensional environments. Unlike other leukocytes, which exclusively used proteinase‐independent amoeboid migration, monocytes and macrophages use either amoeboid migration or the proteinase‐dependent mesenchymal migration mode depending on the density of the tissue to be traversed (Cougoule et al. 2012; Van Goethem et al. 2010). Monocytes and macrophages employ amoeboid migration when migrating through porous matrices but switch to the mesenchymal mode, which involves (and correlates) with the protrusion of podosomes and proteinase‐mediated proteolysis of ECM proteins in more dense matrices such as Matrigel (Cougoule et al., 2012; Van Goethem et al., 2010). However, the proteinases involved in the mesenchymal mode of monocyte and macrophage migration have not been identified.

Herein, we show that Mmp‐8 is not required for monocytes to migrate into a serosal cavity (the peritoneum) as WT and *Mmp*‐*8* ^−/−^ mice with acute sterile peritonitis had similar numbers of macrophages accumulating in their peritoneal cavities. It is noteworthy that prior studies reported that macrophage accumulation in the lungs following LPS and bleomycin‐mediated lung injury was greater in *Mmp*‐*8* ^−/−^ versus WT mice (Craig et al., 2013; Quintero et al., 2010). Collectively, these results indicate that Mmp‐8 is not required for cells to migrate into serosal sacs or porous organs during inflammatory responses consistent with the proteinase‐independent amoeboid macrophage migration mode dominating in these model systems. However, Mmp‐8 contributed significantly to macrophage migration through dense type I collagen gels, layers of SMCs, and the extracellular matrix that they deposited, as *Mmp*‐*8* ^−/−^ macrophages had significantly impaired migration through these tissue barriers when compared with WT macrophages. The Mmp‐8‐mediated surface type I collagenase activity on LPS‐activated macrophages likely contributes to the pericellular proteolysis that was required for these cells to penetrate these dense tissue barriers. As expected, Mmp‐8 was less crucial for macrophage migration through Matrigel and layers of SMC and their associated extracellular matrix, which both contain less type I collagen. It is noteworthy that *Mmp*‐*8* ^−/−^ mice have reduced accumulation of macrophages in experimental atheromatous plaques and an increase in the collagen content of these plaques compared with WT mice (Laxton et al., 2009). These results suggest that macrophage‐derived Mmp‐8 (and likely surface‐bound MMP‐8) is required for macrophage accumulation and contributes to interstitial collagen degradation in atheromatous plaques.

Although MMP‐8 has not previously been implicated in the migration of leukocytes, other MMPs participate in cellular migration. MMP‐1 binds to the α2β1 integrin on the surface of keratinocytes, which confines this proteinase to points of keratinocyte contact with collagen to drive keratinocyte migration. MMP‐2 localizes on the surface of endothelial cells and tumor cells by binding to the α_v_β_3_ integrin (Brooks et al., 1996) or forming a ternary complex with TIMP‐2 bound to MT1‐MMP (Haas et al., 1998) to promote the migration of these cells. However, MMPs (including MMP‐8) indirectly regulate leukocyte migration and accumulation at sites of inflammation by proteolytically activating cytokines and chemokines or generating fragments of ECM (matrikines) that promote the migration of leukocytes into tissues (Churg et al., 2002; Gaggar et al., 2008; Houghton et al., 2006).

TIMPs are efficient inhibitors of soluble forms of MMPs released into extracellular fluids (Owen & Campbell, 1999). However, membrane‐bound TIMP‐1 on PMNs serves as the receptor for MMP‐8 and MMP‐9 on PMNs and thereby promotes pericellular proteolysis (Hirao et al., 2006). We report for the first time that TIMP‐1 is also expressed on the surface of LPS‐activated macrophages. Although we initially hypothesized that Timp‐1 secreted by murine macrophages would bind to the macrophage surface and serve as the anchor for Mmp‐8 on macrophages, our data did not support this hypothesis. It is important to note that the *Timp*‐*1* ^−/−^ murine line that we studied has been confirmed to lack Timp‐1 transcripts and/or protein in cells isolated from different tissues (Kim et al., 2001; Lee et al., 2005; Mohammed et al., 2005; Wang et al. & Owen, 2019). The functions of Timp‐1 on the surface of activated murine macrophages are not clear. However, TIMP‐1 binds to CD63 to promote the survival of myeloid leukocytes by activating focal adhesion kinase (FAK), PI3‐kinase, and ERK (Forte et al., 2017; Kobuch et al., 2015; Liu et al., 2003; Ries, 2014) and has an anti‐apoptotic effect on erythroid cell surfaces by forming a complex with proMMP‐9/CD44 (Lambert et al., 2009). Moreover, murine Timp‐1 increases p53, p57, and p21 protein expression to induce the quiescence of murine hematopoietic stem cells to reduce the number of resident bone marrow cells (Rossi et al., 2011). Although the expression of TIMP‐1 by macrophages is increased in some lung diseases (Manoury et al., 2006) but decreased in other lung diseases (Pons et al., 2005), the contributions of TIMP‐1 to the macrophage function are not known but will be the focus of our future studies.

Other MMPs lacking a transmembrane domain localize on cell surfaces by interacting with bilipids in plasma membranes. Mmp‐12 associates with lipid bilayers on a murine macrophage cell line via basic and hydrophobic residues located in its β‐sheet (Koppisetti et al., 2014). Pro‐MMP‐7 interacts with the lipid bilayer on a human colonic cell line, leading to a conformational change in the MMP inducing its allosteric activation (Prior et al., 2015). MMP‐8 may bind to monocyte and macrophage surfaces by binding lipid bilayers, lipid rafts, or another membrane protein such as an immunoglobulin or an integrin.

A limitation of this study is that we did not determine whether other MMPs lacking a transmembrane domain that are expressed by macrophages (including MMP‐9 and MMP‐12) also bind to the surface of LPS‐activated macrophages. We also did not identify the molecule(s) on the surface of macrophages to which Mmp‐8 binds. These areas will be the focus of our future studies. In addition, we did not purify plasma membranes from activated macrophages and analyze them for the presence and forms of MMP‐8 protein as we have done previously for human peripheral blood PMNs (Owen et al., 2004), which can easily be isolated in the very large cell (>10^8^) numbers needed for these experiments, as it is challenging to isolate this number of macrophages from mice. Nevertheless, we used multiple methods to confirm that MMP‐8 is present on the surface of activated monocytes and macrophages and that it is catalytically active. These complementary methods included immunostaining of MNDs and macrophages and confocal microscopy, surface biotinylation of MDMs, immunoprecipitation of surface biotinylated proteins, and immunoblotting for MMP‐8 and measuring the surface type I collagenase activity associated with fixed LPS‐activated macrophages from WT and *Mmp*‐*8* ^−/−^ mice and also on the surface of unstimulated WT murine macrophages to which we bound exogenous active murine Mmp‐8. We also did not evaluate the Proteolytic Activity Matrix Analysis (PrAMA) method, which is a newer technique (involving integrated experimental measurements and a mathematical analysis framework) that can simultaneously measure the activities of individual enzymes in complex solutions of MMPs (Miller et al., 2011). Whether this method will be sufficiently selective to measure individual MMPs among all of the metalloproteinases present on macrophage surfaces remains to be determined. In addition, our collagenase activity methods used acid‐solubilized type I collagen, which is likely to contain tropocollagen monomers that are more readily degraded than the mature cross‐linked polymeric type I collagen found in tissues.

In conclusion, we report for the first time that MMP‐8 is localized on the surface of LPS‐activated MDM and macrophages where it contributes to pericellular proteolysis. In addition, we identify Mmp‐8 as being a proteinase involved in the macrophage proteinase‐dependent penetration of dense type I collagen‐containing tissue barriers. Thus, surface‐bound MMP‐8 on LPS‐activated monocytes and macrophages could be targeted therapeutically to decrease macrophage tissue infiltration in diseases in which macrophages promote disease progression including idiopathic pulmonary fibrosis and hepatic cirrhosis (Baig et al., 2016; Craig et al., 2014; Tacke, 2017).

## Conflict of interest

Dr. Owen is an employee of Vertex Pharmaceuticals Inc. but has no conflicts of interest relevant to the manuscript to disclose. None of the other authors have any conflicts of interest.

## Authors contribution

CAO and CMD designed the project, contributed to data analysis, and reviewed the manuscript. CMD, CAO, XW, DZ, and QAF conducted experiments. XW and CAO contributed to data analysis and interpretation. XW, CDM, and CAO wrote the manuscript. All authors reviewed and approved the manuscript.
